# Advances in Saponin Diversity of *Panax ginseng*

**DOI:** 10.3390/molecules25153452

**Published:** 2020-07-29

**Authors:** Xiangmin Piao, Hao Zhang, Jong Pyo Kang, Dong Uk Yang, Yali Li, Shifeng Pang, Yinping Jin, Deok Chun Yang, Yingping Wang

**Affiliations:** 1 State Local Joint Engineering Research Center of Ginseng Breeding and Application, Jilin Agriculture University, Changchun 130118, China; pxm52_@163.com (X.P.); zhanghaoscience@163.com (H.Z.); 2 Institute of Special Wild Economic Animals and Plants, Chinese Academy of Agricultural Sciences, Changchun 130112, China; yalilee@126.com (Y.L.); shifengp@126.com (S.P.); jinyinping06@163.com (Y.J.); 3 Graduate School of Biotechnology, College of Life Sciences, Kyung Hee University, Yongin si, Gyeonggi do 17104, Korea; kangjongpyo@naver.com (J.P.K.); rudckfeo23@naver.com (D.U.Y.)

**Keywords:** ginsenoside, *Panax ginseng*, chemical structure, tissue spatial distribution

## Abstract

Ginsenosides are the major bioactive constituents of *Panax ginseng*, which have pharmacological effects. Although there are several reviews in regards to ginsenosides, new ginsenosides have been detected continually in recent years. This review updates the ginsenoside list from *P. ginseng* to 170 by the end of 2019, and aims to highlight the diversity of ginsenosides in multiple dimensions, including chemical structure, tissue spatial distribution, time, and isomeride. Protopanaxadiol, protopanaxatriol and C17 side-chain varied (C17SCV) manners are the major types of ginsenosides, and the constitute of ginsenosides varied significantly among different parts. Only 16 ginsenosides commonly exist in all parts of a ginseng plant. Protopanaxadiol-type ginsenoside is dominant in root, rhizome, leaf, stem, and fruit, whereas malonyl- and C17SCV-type ginsenosides occupy a greater proportion in the flower and flower bud compared with other parts. In respects of isomeride, there are 69 molecular formulas corresponding to 170 ginsenosides, and the median of isomers is 2. This is the first review on diversity of ginsenosides, providing information for reasonable utilization of whole ginseng plant, and the perspective on studying the physiological functions of ginsenoside for the ginseng plant itself is also proposed.

## 1. Introduction

*Panax ginseng* Meyer (*P. ginseng*), known as the king of all herbs, has been frequently used as traditional medicine and healthy food in China, Korea, and Japan. In 2012, *P. ginseng* was approved as a new food resource by Chinese government, and it has been widely used as the raw material of healthcare products [1]. Ginseng contains a large amount and number of ginsenosides. More than 289 saponins were reported from eleven different *Panax* species [2]. In addition, at least 123 ginsenosides have been identified in different *P. ginseng* species, and these include both naturally occurring compounds and those from steaming and biotransformation [3]. In addition, 112 saponins were reported from raw or processed ginseng, including hydrolysates, semisynthetic, and metabolites [4]. Ginsenosides are known to possess a lot of biological activities including regulatory effects on immunomodulation, protection functions in the central nervous and cardiovascular systems, anti-diabetic, anti-aging, anti-carcinogenic, anti-fatigue, anti-pyretic, anti-stress, boosting physical vitality, and promotion of DNA, RNA, and protein synthesis activities [5,6,7,8,9]. In addition, the biosynthesis of triterpenoid is an important factor of saponin diversity. Consequently, biosynthetic mechanisms for the backbone synthesis [4] and structural diversification and genes/enzymes involved in the biosynthesis [10] were reviewed in the cited references. Therefore, ginsenosides are recognized as the main bioactive components and a key index for quality evaluation of ginseng.

Due to the complexity of the ginsenosides and their structures, multi-platform analytical techniques are used in the detection of ginseng products, such as thin layer chromatography (TLC), high performance thin layer chromatography (HPTLC), gas chromatography (GC), high performance liquid chromatography (HPLC), ultra performance liquid chromatography (UPLC) [3,11,12]. However, these methods detect only small numbers of ginsenosides and lack in provision of structural information. Liquid chromatography coupled with tandem mass spectrometry can provide structural information with high sensitivity, specificity, and versatility in characterizing complex natural product samples. It has been successfully used as a powerful tool for ginsenoside analysis with high throughput [1]. In recent years, a number of novel ginsenosides have been detected in aerial parts of the ginseng plant using the HPLC-MS/MS method, such as stems, leaves, rhizomes, flowers, and flower buds, which enlarged the number of ginsenoside family members [13,14,15]. Several reviews have summarized the progress from a viewpoint of structural features, and conclude that ginsenosides are generally classified into four groups: protopanaxadiol type (PPD), protopanaxatriol type (PPT), C17 side-chain varied type (C17SCV), and oleanolic acid type (OA) [2,16,17,18]. However, spatial distribution of ginsenoside in different parts of *P. ginseng* is not yet summarized. This information will make better use of the whole ginseng plant and provide clues for studying the biological function of saponins. This review updates the ginsenoside list (from *P. ginseng*) to 170 by the end of 2019, and aims to highlight the diversity of ginsenosides in multiple dimensions, including chemical structure, tissue spatial distribution, time, and isomeride.

## 2. History of Saponins Isolated from *P. ginseng*

The history of ginsenoside isolation can be divided into three periods (before 1980 for Period I, 1980–2000 for Period II, after 2000 for Period III) based on the development of analytical techniques. The study on ginsenoside started in 1854. A ginsenoside-containing constituent was firstly isolated from American ginseng by American scholar Garriques [19], and subsequently, Japanese chemists reported panaquilon, panacon, panaxasapogenol, and ginsenin preliminarily separated from *P. ginseng*. For almost 100 years since the middle of the nineteenth century, it was difficult to obtain a pure ginsenoside due to the under development of separation techniques. In the early 1950s, with the development of separation technology and the invention of modern analytical instruments, such as GC, TLC, etc., the studies on the chemical ingredient of ginseng made remarkable progress. In 1963, for the first time, Shibata et al. reported the chemical property and structure of the panaxadiol separated from ginseng root [20]. In the 1970s, 17 ginsenosides were detected in ginseng, named as ginsenoside Ro, Ra, Rb1, Rb2, Rc, Rd, Re, Rf, Rg1, Rg2, Rg3, F1, F2, F3, Rb3, Rh, and 20-glucoginsenoside-Rf [21,22,23,24,25,26]. The second period began when the ^13^C NMR technique was introduced into the structure analysis of ginsenosides. By comparison of the measured ^13^C NMR spectroscopic data with known compounds, the accurate structure of new ginsenosides (G-Rh1, Rh2, Rh3, Rg4, Ra1, Ra2, Ra3, La, Rf2, Rs3, Ia, Ib, etc.) could be resolved from different parts of ginseng (root, steamed root, flower bud, stem, and leaf). In this period, more and more scientists focused on ginsenoside isolation, and most of ginsenosides were found in the aerial parts of ginseng [27,28,29,30,31,32,33,34,35,36]. The third period was defined by high-efficiency separation methods, as methods such as high-speed counter current chromatography (HSCCC), high performance centrifugal partition chromatography (HPCPC), and 2D NMR spectroscopic techniques were used for separating and identifying ginsenosides. The application of these powerful new techniques helps to identify the complex chemical structure, for instance, C17 side-chain variation and malonyl group. More than 50 new ginsenosides were isolated from 2000 to 2019, among which most of those possessed variations in the C17 side-chain, besides a part of malonyl ginsenosides [37,38,39,40,41].

## 3. Classification of Saponins Identified from *P. ginseng*

**Figure 1:**
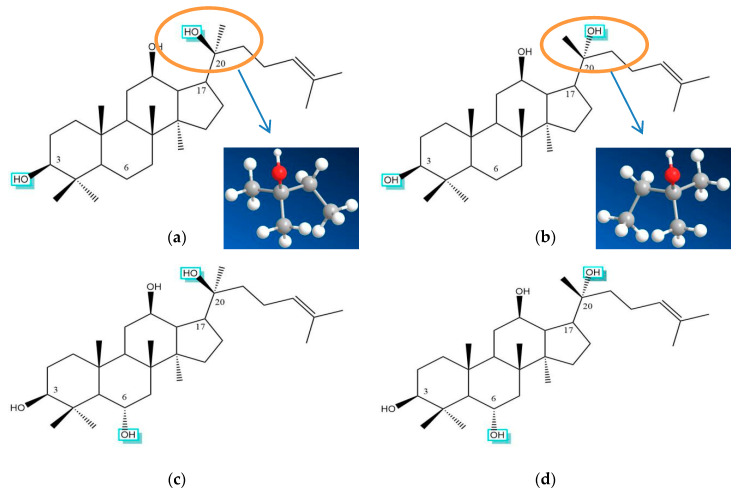

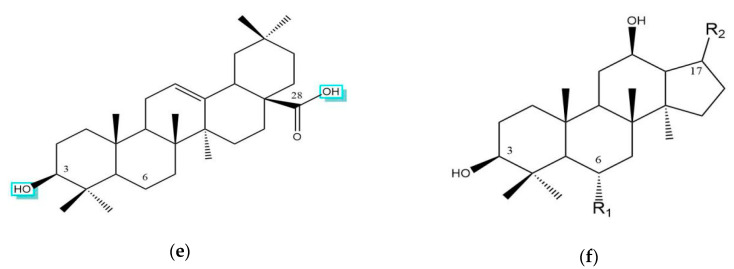
Structures of PPD, PPT, OA, and C17SCV sapogenins. The typical glycosylation sites for these sapogenins are marked in blue frame. (**a**) 20(*S*)-PPD: Protopanaxadiol type; (**b**) 20(*R*)-PPD: Protopanaxadiol type; (**c**) 20(*S*)-PPT: Protopanaxatriol type; (**d**) 20(*R*)-PPT: Protopanaxatriol type; (**e**) OA: Oleanolic acid type; (**f**) C17SCV: C17 side-chain variation type. R1 in C17SCV: -H, -OH, -OR. R2 in C17SCV: The variations in the C17 side-chain mainly comprise H_2_O-addition, hydroxylation, methoxylation, peroxidization, dehydration at C-20, carbonylation, dehydrogenation, cyclization, oxidation (at the double bond), and degradation. The stereochemistry of chiral centers are shown in (**a**) and (**b**).

**Figure 2:**
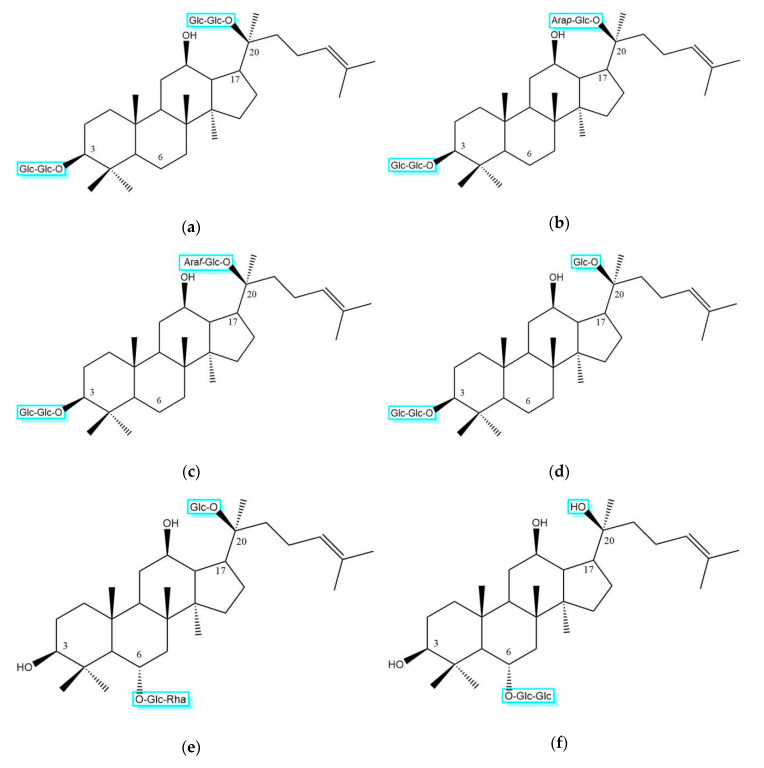

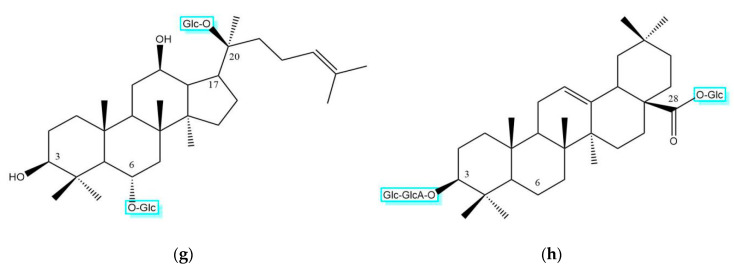
Structures of eight high-abundance saponins in *P. ginseng*. (**a**) PPD-type ginsenoside Rb1; (**b**) PPD-type ginsenoside Rb2; (**c**) PPD-type ginsenoside Rc; (**d**) PPD-type ginsenoside Rd; (**e**) PPT-type ginsenoside Re; (**f**) PPT-type ginsenoside Rf; (**g**) PPT-type ginsenoside Rg1; (**h**) OA-type ginsenoside Ro.

Although most ginsenosides have a rigid four-trans-ring steroid skeleton, they produce multiple pharmacological and biological effects that are different from one another due to minor variations on: (1) Type of sapogenins; (2) number, type, and site of glycosyl units; and (3) modification of C17 side-chains [11,42,43]. Therefore, the study of ginsenoside structure will help to elucidate the mechanism of multiple functions of ginsenosides. The reported ginsenosides are classified into protopanaxadiol type (PPD), protopanaxatriol type (PPT), oleanolic acid type (OA), and C17 side-chain varied (C17SCV) subtypes according to their determined sapogenin structures (Figure 1). The glycosyl components of saponin were mainly β-d-glucopyranosyl group, followed by α-l-rhamnopyranosyl group, a few binding α-l-arabinopyranosyl group and β-d-xylopyranosyl group, and the β-d-glucopyranosiduronyl group only appears in saponins with oleanolic acid-type (OA) sapogenin. In dammarane-type triterpenoid saponins, β-d-glucopyranosyl group (2→1)-β-d-glucopyranosyl oligosaccharide chains occur more frequently, and are mostly bound to C-3 of sapogenin to generate oxyglycoside; β-d-glucopyranosyl group (2→1)→α-l-rhamnopyranosyl group oligosaccharide chains are mostly bound to C-6 of sapogenin to form oxyglycoside. The tetracyclic parent nucleuses are relatively stable, whether they are PPT and/or PPD type. Moreover, the substituents that occur in the C17 side-chains often undergo oxidation, reduction, cyclization, and epimerization, contributing to diversity in chemical structure [12,16]. Table 1 displays the molecular formulas, molecular masses, and structural categories of 170 ginsenosides, isolated from different parts of *P. ginseng*. As a result, four ginsenosides are OA type, 59 ginsenosides are PPD type, 42 ginsenosides are PPT type, and 65 ginsenosides are C17CSV type. Among them, four PPD-type ginsenosides (Rb1, Rb2, Rc, Rd), three PPT-type ginsenosides (Re, Rf, Rg1), and one OA-type ginsenoside Ro (the structures are shown in Figure 2) are the most abundant in *P. ginseng*, and account for more than 70% of the total saponins [5].

## 4. Spatial Distribution of Ginsenosides in Different Parts

**Figure 3:**
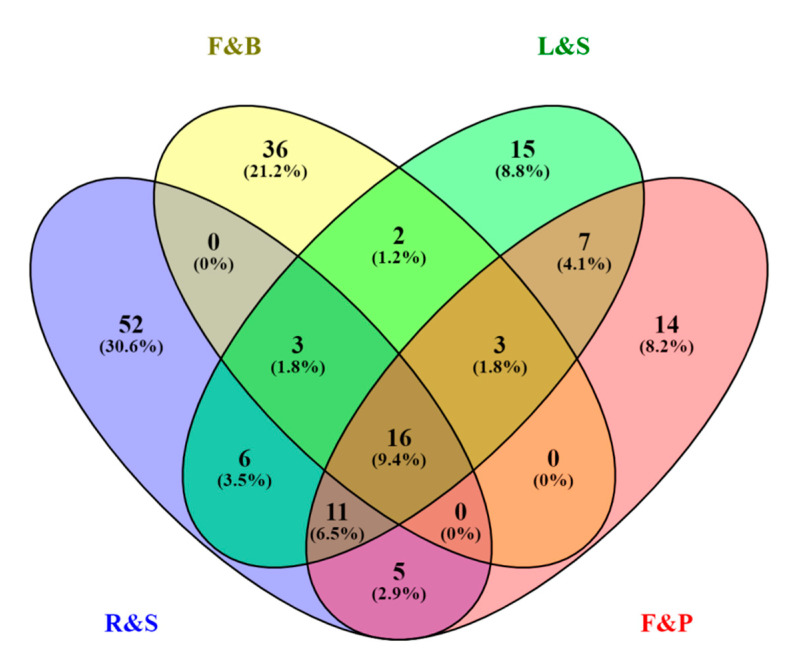
Venn diagram of ginsenosides according to different parts of *P. ginseng*. R&S: Roots, rhizomes, and steamed roots; L&S: Leaves and stems; F&P: Fruits and fruit pedicels; F&B: Flowers and flower buds.

The Venn diagram (Figure 3) shows the number of ginsenosides commonly and separately shared by the following four groups: R&S (roots, rhizomes, and steamed roots), L&S (leaves and stems), F&P (fruits and fruit pedicels), and F&B (flowers and flower buds). Among them, the number of unique ginsenosides in group R&S, F&P, L&S, and F&B are 52, 15, 14, and 36, respectively, accounting for 30.6%, 8.8%, 8.2%, and 21.2% of the number of total ginsenosides, respectively. The result gives some explanation why ginseng root is designated as medicinal parts rather than the other parts. Sixteen ginsenosides are commonly existed in all tissues, and among them, there are nine PPD type (Rc, Rd, Rb2, Rb1, Rb3, m-ginsenoside Rb1, m-ginsenoside Rc, m-ginsenoside Rb2, m-ginsenoside Rd), six PPT type (Re, Rg1, Rf, 20(*R*)-ginsenoside Rg2, Notoginsenoside R1, m-ginsenoside Re), one OA type (Ro), and none of C17SCV type. Numbers of ginsenosides shared by R&S and F&P, F&P and L&S, L&S and F&B, R&S and F&B were 32 (18.8%), 37 (21.7%), 24 (14.1%), and 19(11.2%), respectively. In addition, 13 malonyl-ginsenosides were existing specifically in flowers and buds; however, none of them was observed in fruit. This implies that these malonyl-ginsenosides show not only spatial specificity, but also temporal specificity. Here in, we speculate that malonyl-ginsenosides may play a physiological role during tissue development.

**Figure 4:**
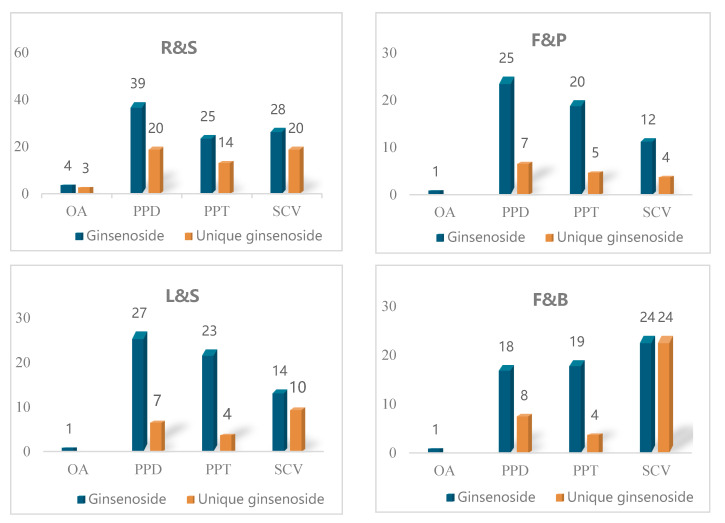
Structural categories of ginsenosides in different parts of *P. ginseng*. R&S: Roots, rhizomes, and steamed roots; F&P: Fruits and fruit pedicels; L&S: Leaves and stems; F&B: Flowers and flower buds; OA: Oleanolic acid; PPD: Protopanaxadiol; PPT: Protopanaxatriol; C17SCV: C17 side-chain varied.

As indicated by Figure 4, the numbers of PPD-type ginsenosides (blue bar) are highest in R&S, F&P, and L&S, while the C17SCV-type ginsenoside is highest in F&B. Interestingly, C17SCV-type ginsenosides exhibit significant variation among different groups. Only nine C17SCV-type ginsenosides are shared by more than two groups, whereas the other 58 C17SCV-type ginsenosides are unique to a particular group. For the OA-type ginsenoside, three are specific to group R&S (Polyacetyleneginsenoside-Ro, Ginsenoside Ro methyl ester, Calenduloside-B) and one (Ginsenoside Ro) is commonly shared by all parts.

## 5. Isomers of Ginsenosides

The total 170 ginsenosides are divided into 69 molecular formula groups. Therefore, it is common that one molecular formula corresponds to several ginsenosides. (Table 2). The molecular formula with the largest number of isomers is C_48_H_82_O_19_ (molecular weight 962.5450), with a total of nine isomers; followed by C_51_H_84_O_21_ (molecular weight 1032.5505) with a total of eight isomers, and C_41_H_70_O_13_ (molecular weight 770.4816) with a total of seven isomers. The isomers median of 69 molecular formulas is 2, which means that one molecular formula corresponds to two isomers equally. Optical and position isomerism are the dominant types of ginsenoside isomers, whilst cis-trans isomerism and tautomerism are detected occasionally.

## 6. Mass Spectrometry-Based Metabolomics Analysis on *P. ginseng*

Recently, MS and its hyphenations with chromatographic separation techniques have emerged as an instrumental trend in ginsenoside analysis [93,94]. HPLC/MS can overcome the problems related to ginsenoside pre-analysis derivatization and the low abundance of molecular ions [95,96]. The use of on-line MS detection shows superior sensitivity and specificity compared with conventional UV and ELSD detection [97,98]. The sensitivity of MS detection can surpass 1000 times that of UV absorbance [99]. In addition, the possible matrix effects encountered with many *Panax ginseng* formulations may be compromised by MS [100]. Despite these advantages, MS remains costly for use in routine analysis. With the development of soft ionization techniques, HPLC/MS has been successfully applied for the qualitative and quantitative analyses of *Panax ginseng* [101]. Among the various mass spectrometry ionization techniques, electrospray mass spectrometry (ESI-MS) is the approach that is most commonly coupled with HPLC [15,102,103]. While ESI-MS suffers from matrix-induced ionization suppression difficulties [104], atmospheric pressure chemical ionization (APCI) can offer itself as one possible alternative [105]. Quadrupole time-of-flight mass spectrometry (QTOF-MS), a powerful tool for the identification of analytes, provides several advantages in structural analysis, such as a higher resolution and accuracy in mass measurements. Coupled with QTOF-MS, UPLC has been introduced for metabolite profiling and metabolomics purposes [99]. In recent years, orbitrap technology has achieved great breakthrough in resolution and scanning speed and realized the high-resolution detection of multi-stage mass spectrometry by combining the linear ion trap and quadrupole mass spectrometry, which can be widely applied in the development of new drugs [106].

According to the available literature, Wang et al. in 1999 [97] firstly identified ginsenosides by LC/MS/MS and differentiated *P. ginseng* and *P. quinquefolius* based on the ginsenoside Rg1/Rf and Rc/Rb2 ratios. A liquid chromatography-tandem mass spectrometry (LC/MS/MS) method was developed to distinguish Asian ginseng and North American ginseng. The method is based on the baseline chromatographic separation of two potential chemical markers: Rf and 24(*R*)-pseudo ginsenoside F11 [107]. Z X. et al. 2000 developed a similar LC/MS/MS method to determine ginsenoside in ginseng. Nine ginsenosides were determined, among which five of them were identified according to molecular weight [108]. In the late 1990s and early 2000s, the resolution of mass spectrometry was low and the number of identified ginsenosides was limited, which could be used for distinguishing Asian ginseng and American Ginseng, and identifying ginsenosides.

Chen et al. [109] established a chemical finger-print metabolomics approach using ultra-high-performance liquid chromatography combined with quadrupole time-of-flight mass spectrometry (UPLC-QTOF/MS). The method was successfully used to authenticate and evaluate *Panax Ginseng* of various commercial grades. Using UPLC-QTOF-MS/MS, Zhang et al. evaluated the overall quality of commercially available white ginseng and red ginseng, and investigated their characteristic chemical composition indicators. Fifty-one major chromatographic peaks of white ginseng and red ginseng samples were separated within 24 min [110]. By means of UPLC-DAD-QTOF-MS/MS, Wang et al. conducted qualitative and quantitative analysis of ginsenosides of cultivated ginseng and mountain ginseng. A total of 131 ginsenosides were detected in cultivated ginseng and mountain ginseng, and all the components were completely separated within 10 min, among which contents of 19 typical ginsenoside were accurately quantified. This method has been validated for quality evaluation of ginseng and identification of cultivated ginseng and mountain ginseng [13]. Zhang et al. Quickly and comprehensively identified the ginsenosides using high-resolution time-of-flight mass spectrometry, electrospray dual-spray ion source, and negative ion mode. A total of 95 saponins in suncured ginseng were identified within 11 min, providing a feasible basis for the quality control of suncured ginseng [111]. With the emergence of high-resolution mass spectrometry and the development of high-throughput screening technologies, several time-saving methods were established for commercial ginseng product evaluation.

Since 2015, Orbitrap mass spectrometer had been applied in ginsenoside detection. In 2017, a total of 101 malonyl-ginsenosides were firstly systematic analyzed by hybrid LTQ-Orbitrap mass spectrometer after UHPLC separation, and ten potential malonyl-ginsenoside markers were discovered for the discrimination of *P. ginseng*, *P. quinquefolius*, and *P. notoginseng* [112]. Shi et al. established an untargeted profiling strategy on a linear ion-trap/Orbitrap mass spectrometer coupled to ultra-high performance liquid chromatography to analyze malonyl-ginsenosides in several *Panax* species. Finally, 178 malonyl-ginsenosides were characterized from roots, leaves, and flower buds of *P. ginseng*, *P. quinquefolius*, and *P. notoginseng* [113]. To investigate the variation of ginsenosides among different processed red ginseng, Zhong et al. tested steamed, vinegared and dried red ginseng samples by UPLC-Q-Orbitrap MS. In total, 32 ginsenosides were identified and ginsenosides m-Rb1, Rh1, F1, 20(*R*)-Rh1, Rg5, and Rs5 were only found in red ginseng processed by vinegar [114]. With the development of Orbitrap and multi-mass spectrometry techniques, ginsenosides with complex structures, such as malonyl and C17 side-chain variation, have been increasingly detected, and the types of ginsenosides have been greatly extended.

## 7. Conclusions

In this review, we summarized the existing studies related to saponin analysis of *P. ginseng*, and sorted out the information of structural characteristic, spatial distribution, and isomer of 170 ginsenosides. There are 16 common ginsenosides present in all parts of *P. ginseng*. In contrast, each part has unique ginsenosides, and ginsenosides in different parts show obvious structural diversity. It should be emphasized that ginseng aerial parts can regenerate every year, and there is a large amount of rare ginsenosides in stems, leaves, and flower buds. In light of previous research results of the rare ginsenoside bioactivity in red ginseng, it seems that the aerial parts of *P. ginseng* are highly worth developing and utilizing. A conclusion can also be drawn that C17SCV-type ginsenosides and malonyl-ginsenoside are rich in flowers and buds. Therefore, a hypothesis that ginsenosides have physiological roles in ginseng plant development is proposed. The rapid development of high-performance liquid chromatography and mass spectrometry techniques significantly raise the throughput and accuracy of ginsenoside determination.

In the future, (1) with the continuous advancement of detection and identification technology, the analysis method of ginsenosides will develop in the direction of being more sensitive, convenient, and environmentally-friendly, with high-throughput and high-precision. By leveraging these technologies, more monomer compounds will be separated and identified from ginseng, which will develop the knowledge of the diversity of chemical structure of ginsenosides. (2) It is necessary to conduct further research on spatial distribution of ginsenosides in different parts of ginseng, and multidisciplinary collaborations among genomics, proteomics, metabonomics, and transcriptomics could be used to study the physiological functions of ginsenosides. (3) With increasing separation of ginsenosides possessing a complex structure, such as malonyl and C17 side-chain variation, the pharmacological action and pharmacokinetics of these ginsenosides would be further studied to clarify the efficacy of ginseng.

## Figures and Tables

**Table 1:** The 170 ginsenosides isolated from *P. ginseng.*

No.	Subtype	Saponins	Formula	Molecular Mass	Plant Part	Refs
1	OA ^1^	Polyacetylene ginsenoside Ro	C_65_H_100_O_21_	1216.6757	Root	[44]
2	OA	Ginsenoside Ro methyl ester	C_49_H_78_O_19_	970.5137	Root(steamed)	[45]
3	OA	Calenduloside B	C_48_H_78_O_18_	942.5188	Root	[46]
4	OA	Ginsenoside Ro	C_49_H_80_O_18_	956.5345	Root, flower, fruit, leaf	[16,47]
5	PPD	Ginsenoside Ra1	C_58_H_98_O_26_	1210.6346	Root	[48]
6	PPD	Ginsenoside Ra2	C_58_H_98_O_26_	1210.6346	Root	[49]
7	PPD	Ginsenoside Ra3	C_59_H_100_O_27_	1240.6452	Root	[50]
8	PPD	Ginsenoside Rs1	C_55_H_92_O_23_	1120.6029	Root(steamed)	[51]
9	PPD	Ginsenoside Rs2	C_55_H_92_O_23_	1120.6029	Root(steamed)	[51]
10	PPD	Malonyl-ginsenoside Ra3	C_62_H_102_O_30_	1326.6456	Root(fresh)	[52]
11	PPD	Malonyl-notoginsenoside R4	C_62_H_102_O_30_	1326.6456	Root	[52]
12	PPD	Ginsenoside Ra4	C_62_H_102_O_27_	1278.6608	Root	[53]
13	PPD	Ginsenoside Ra5	C_60_H_99_O_27_	1251.6373	Root	[53]
14	PPD	Ginsenoside Ra6	C_58_H_96_O_24_	1176.6292	Root	[53]
15	PPD	Ginsenoside Ra7	C_57_H_93_O_23_	1145.6108	Root	[53]
16	PPD	Ginsenoside Ra8	C_57_H_94_O_23_	1146.6186	Root	[53]
17	PPD	Ginsenoside Ra9	C_57_H_94_O_23_	1146.6186	Root	[53]
18	PPD	20(*S*)-ginsenoside Rg3	C_42_H_72_O_13_	784.4973	Root(steamed), fruit, leaf	[54]
19	PPD	Ginsenoside Rs3	C_44_H_74_O_14_	826.5079	Root(steamed)	[55]
20	PPD	Ginsenoside IV	C_58_H_96_O_24_	1176.6292	Root	[47]
21	PPD	Ginsenoside V	C_54_H_92_O_24_	1124.5979	Root	[47]
22	PPD	Gypenoside-V	C_54_H_92_O_22_	1092.6080	Root	[46]
23	PPD	20(*R*)-ginsenoside Rs3	C_44_H_74_O_14_	826.5079	Root(steamed)	[45]
24	PPD	Acetyl-ginsenoside Rd	C_50_H_84_O_19_	988.5607	Root(mountain ginseng)	[56]
25	PPD	Ginsenoside F2	C_42_H_72_O_13_	784.4973	Root, fruit, leaf	[57]
26	PPD	Pseudoginsenoside Rc1	C_50_H_84_O_19_	988.5607	Fruit	[57]
27	PPD	Gypenoside XVII	C_48_H_82_O_18_	946.5501	Fruit, leaf	[57]
28	PPD	Gypenoside IX	C_47_H_80_O_17_	916.5396	Fruit, leaf	[57]
29	PPD	Quinquenoside L10	C_47_H_80_O_17_	916.5396	Fruit	[57]
30	PPD	25-Hydroxyprotopanaxadiol	C_30_H_54_O_4_	478.4022	Fruit	[58]
31	PPD	20(*S*)-protopanaxadiol	C_30_H_52_O_3_	460.3916	Fruit, leaf	[41,59]
32	PPD	20(*R*)-protopanaxadiol	C_30_H_52_O_3_	460.3916	Fruit	[59]
33	PPD	Notoginsenoside Fd	C_47_H_80_O_17_	916.5396	Fruit	[60]
34	PPD	Ginsenoside Rd2	C_47_H_80_O_17_	916.5396	Leaf	[61]
35	PPD	20(*R*)-ginsenoside Rg3	C_42_H_72_O_13_	784.4973	Root(steamed), fruit, leaf	[62,63]
36	PPD	20(*S*)-ginsenoside Rh2	C_36_H_62_O_8_	622.4445	Root(steamed), fruit, leaf	[64]
37	PPD	20(*R*)-ginsenoside Rh2	C_36_H_62_O_8_	622.4445	Fruit, leaf	[65]
38	PPD	Notoginsenoside Fe	C_47_H_80_O_17_	916.5396	Fruit, leaf	[61]
39	PPD	Acetyl-ginsenoside Rb1	C_56_H_96_O_24_	1152.6292	Root(mountain ginseng), leaf	[56]
40	PPD	Acetyl-ginsenoside Rc	C_55_H_92_O_23_	1120.6029	Root(mountain ginseng), leaf	[56]
41	PPD	Acetyl-ginsenoside Rb3	C_55_H_92_O_23_	1120.6029	Root(mountain ginseng), leaf	[56]
42	PPD	Ginsenoside compound O	C_47_H_80_O_17_	916.5396	Root, fruit, leaf	[16,66]
43	PPD	Malonyl-ginsenoside Rb2	C_56_H_92_O_25_	1164.5928	Root, flower, fruit, leaf	[16]
44	PPD	Ginsenoside Mc	C_41_H_70_O_12_	754.4867	Leaf	[16,66]
45	PPD	Ginsenoside compound Y	C_41_H_70_O_12_	754.4867	Leaf	[16]
46	PPD	Ginsenoside compound K	C_36_H_62_O_8_	622.4445	Root, fruit, leaf	[16]
47	PPD	Ginsenoside Rb1	C_54_H_92_O_23_	1108.6029	Root, flower, fruit, leaf	[16,67]
48	PPD	Malonyl-ginsenoside Rb1	C_57_H_94_O_25_	1178.6084	Root, flower, fruit, leaf	[16,67]
49	PPD	Ginsenoside Rc	C_53_H_90_O_22_	1078.5924	Root, flower, fruit, leaf	[16,67]
50	PPD	Malonyl-ginsenoside Rc	C_56_H_92_O_25_	1164.5928	Root, flower, fruit, leaf	[16,67]
51	PPD	Ginsenoside Rb2	C_53_H_90_O_22_	1078.5924	Root, flower, fruit, leaf	[16,67]
52	PPD	Ginsenoside Rb3	C_53_H_90_O_22_	1078.5924	Root, flower, fruit, leaf	[16,67]
53	PPD	Malonyl-ginsenoside Rb3	C_56_H_92_O_25_	1164.5928	Root, flower, leaf	[16,67]
54	PPD	Ginsenoside Rd	C_48_H_82_O_18_	946.5501	Root, flower, fruit, leaf	[16,67]
55	PPD	Malonyl-ginsenoside Rd	C_51_H_84_O_21_	1032.5505	Root, flower, fruit, leaf	[16,67]
56	PPD	Malonyl-floralginsenoside Rd2	C_51_H_84_O_21_	1032.5505	Flower	[68]
57	PPD	Malonyl-floralginsenoside Rd3	C_51_H_84_O_21_	1032.5505	Flower	[68]
58	PPD	Malonyl-floralginsenoside Rd4	C_51_H_84_O_21_	1032.5505	Flower	[68]
59	PPD	Malonyl-floralginsenoside Rd5	C_51_H_84_O_21_	1032.5505	Flower	[68]
60	PPD	Malonyl-floralginsenoside Rd6	C_54_H_87_O_24_	1119.5587	Flower	[68]
61	PPD	Malonyl-floralginsenoside Rc2	C_56_H_92_O_25_	1164.5928	Flower	[68]
62	PPD	Malonyl-floralginsenoside Rc3	C_56_H_92_O_25_	1164.5928	Flower	[68]
63	PPD	Malonyl-floralginsenoside Rc4	C_56_H_92_O_25_	1164.5928	Flower	[68]
64	PPT	20(*S*)-ginsenoside Rg2	C_42_H_72_O_13_	784.4973	Root, fruit, leaf	[54,69]
65	PPT	Koryoginsenoside R1	C_46_H_76_O_15_	868.5184	Root	[36]
66	PPT	Ginsenoside Re6	C_46_H_76_O_15_	868.5184	Root	[70]
67	PPT	Ginsenoside Re2	C_48_H_82_O_19_	962.5450	Root	[70]
68	PPT	Ginsenoside Re3	C_48_H_82_O_19_	962.5450	Root	[70]
69	PPT	Ginsenoside Re4	C_47_H_80_O_18_	932.5345	Root	[70]
70	PPT	Notoginsenoside Rt	C_44_H_74_O_15_	842.5028	Root	[46]
71	PPT	Majoroside F6	C_48_H_82_O_19_	962.5450	Root	[46]
72	PPT	Pseudoginsenoside Rt3	C_42_H_70_O_13_	782.4816	Root	[46]
73	PPT	Vinaginsenoside R15	C_42_H_72_O_15_	816.4871	Root	[46]
74	PPT	20(*R*)-ginsenoside Rf	C_42_H_72_O_14_	800.4922	Root	[45]
75	PPT	20(*R*)-notoginsenoside R2	C_41_H_70_O_13_	770.4816	Root	[45]
76	PPT	Ginsenoside Ia	C_42_H_72_O_14_	800.4922	Fruit	[71]
77	PPT	Chikusetsusaponin LM1	C_41_H_70_O_13_	770.4816	Fruit	[57]
78	PPT	25-Hydroxyprotopanaxatriol	C_30_H_54_O_5_	494.3971	Fruit	[58]
79	PPT	20(*S*)-protopanaxatriol	C_30_H_52_O_4_	476.3866	Fruit, leaf	[59]
80	PPT	20(*R*)-protopanaxatriol	C_30_H_52_O_4_	476.3866	Fruit	[59]
81	PPT	Notoginsenoside R3	C_48_H_82_O_19_	962.5450	Fruit	[60]
82	PPT	20-glucoginsenoside Rf	C_48_H_82_O_19_	962.5450	Root, flower, leaf	[16]
83	PPT	Saponin IIb	C_36_H_62_O_9_	638.4394	Leaf	[72]
84	PPT	Saponin IIIc	C_37_H_62_O_10_	666.4343	Leaf	[72]
85	PPT	20(*S*)-ginsenoside Rh1	C_36_H_62_O_9_	638.4394	Leaf	[62]
86	PPT	20(*R*)-ginsenoside Rh1	C_36_H_62_O_9_	638.4394	Root(steamed), leaf	[21]
87	PPT	Acetyl-ginsenoside Rg1	C_44_H_74_O_15_	842.5028	Root(mountain ginseng), leaf	[56]
88	PPT	Acetyl-ginsenoside Re	C_50_H_84_O_19_	988.5607	Root(mountain ginseng), leaf	[56]
89	PPT	Notoginsenoside R2	C_41_H_70_O_13_	770.4816	Root, fruit, leaf	[16]
90	PPT	Notoginsenoside R1	C_47_H_80_O_18_	932.5345	Root, flower, fruit, leaf	[16,67]
91	PPT	Ginsenoside Rg1	C_42_H_72_O_14_	800.4922	Root, flower, fruit, leaf	[16,67]
92	PPT	Ginsenoside Re	C_48_H_82_O_18_	946.5501	Root, flower, fruit, leaf	[16,67]
93	PPT	Malonyl-ginsenoside Rg1	C_45_H_74_O_17_	886.4926	Root, flower, leaf	[16,67]
94	PPT	Malonyl-ginsenoside Re	C_51_H_84_O_21_	1032.5505	Root, flower, fruit, leaf	[16,67]
95	PPT	Ginsenoside Rf	C_42_H_72_O_14_	800.4922	Root, flower, fruit, leaf	[16,67]
96	PPT	20(*R*)-ginsenoside Rg2	C_42_H_72_O_13_	784.4973	Root(steamed), flower, fruit, leaf	[16,67]
97	PPT	Ginsenoside Rf3	C_41_H_70_O_13_	770.4816	Flower	[67]
98	PPT	Floralginsenoside M	C_53_H_90_O_22_	1078.5924	Flower, leaf	[73]
99	PPT	Floralginsenoside N	C_53_H_90_O_22_	1078.5924	Flower, leaf	[73]
100	PPT	Floralginsenoside P	C_53_H_90_O_23_	1094.5873	Flower	[73]
101	PPT	Ginsenoside F1	C_36_H_62_O_9_	638.4394	Flower, fruit, leaf	[74]
102	PPT	Ginsenoside F3	C_41_H_70_O_13_	770.4816	Flower, fruit, leaf	[74]
103	PPT	Ginsenoside F5	C_41_H_70_O_13_	770.4816	Flower, fruit, leaf	[74]
104	PPT	Malonyl-floralginsenoside Re2	C_51_H_84_O_21_	1032.5505	Flower	[68]
105	PPT	Malonyl-floralginsenoside Re3	C_51_H_84_O_21_	1032.5505	Flower	[68]
106	C17SCV	Koryoginsenoside R2	C_54_H_92_O_24_	1124.5979	Root	[36]
107	C17SCV	Ginsenoside Re5	C_42_H_72_O_15_	816.4871	Root	[70]
108	C17SCV	Ginsenoside Rs4	C_44_H_72_O_13_	808.4973	Root(sun cured)	[75]
109	C17SCV	Dehydroprotopanaxadiol I	C_30_H_50_O_2_	442.3811	Root(steamed)	[2]
110	C17SCV	Ginsenoside Rg5	C_42_H_70_O_12_	766.4867	Root(steamed)	[76]
111	C17SCV	Dehydroprotopanaxatriol I	C_30_H_50_O_3_	458.3760	Root(steamed)	[2]
112	C17SCV	Ginsenoside Rs6	C_38_H_62_O_9_	662.4394	Root(sun cured)	[75]
113	C17SCV	Ginsenoside Rz1	C_42_H_70_O_12_	766.4867	Root(steamed)	[77]
114	C17SCV	Dehydroprotopanaxadiol II	C_30_H_50_O_2_	442.3811	Root(steamed)	[2]
115	C17SCV	Ginsenoside Rs5	C_44_H_72_O_13_	808.4973	Root(sun cured)	[75]
116	C17SCV	Dehydroprotopanaxatriol II	C_30_H_50_O_3_	458.3760	Root(steamed)	[2]
117	C17SCV	Ginsenoside Rg6	C_42_H_70_O_12_	766.4867	Root(steamed)	[78]
118	C17SCV	Ginsenoside Rk3	C_36_H_60_O_8_	620.4288	Root(steamed)	[76]
119	C17SCV	Ginsenoside Rs7	C_38_H_62_O_9_	662.4394	Root(sun cured)	[75]
120	C17SCV	Ginsenoside Rg9	C_42_H_70_O_13_	782.4816	Root(steamed)	[79]
121	C17SCV	12-*O*-glucoginsenoside Rh4	C_42_H_70_O_13_	782.4816	Root(steamed)	[80]
122	C17SCV	Ginsenoside Rg10	C_42_H_69_O_13_	781.4738	Root(steamed)	[79]
123	C17SCV	Ginsenoside Rh10	C_36_H_62_O_8_	622.4445	Root(steamed)	[80]
124	C17SCV	Ginsenoside Rg11	C_42_H_70_O_14_	798.4766	Root(steamed)	[80]
125	C17SCV	Vinaginsenoside R8	C_48_H_82_O_19_	962.5450	Fruit	[57]
126	C17SCV	Ginsenoside Rh4	C_36_H_60_O_8_	620.4288	Root(steamed), fruit	[4,57]
127	C17SCV	Ginsenoside Rh5	C_36_H_60_O_9_	636.4237	Root(steamed), fruit	[4,57]
128	C17SCV	Isoginsenoside-Rh3	C_36_H_60_O_7_	604.4339	Fruit	[81]
129	C17SCV	Ginsenoside Rf2	C_42_H_72_O_14_	800.4922	Fruit	[82]
130	C17SCV	Ginsenoside Rk2	C_36_H_60_O_7_	604.4339	Root(steamed), fruit	[76,83]
131	C17SCV	Pseudoginsenoside RT5	C_36_H_62_O_10_	654.4343	Fruit	[83]
132	C17SCV	Ginsenoside Rh3	C_36_H_60_O_7_	604.4339	Root(steamed), fruit	[76,83]
133	C17SCV	Ginsenoside Rg4	C_42_H_70_O_12_	766.4867	Root, fruit	[16]
134	C17SCV	Ginsenoside F4	C_42_H_70_O_12_	766.4867	Root, fruit, leaf	[16]
135	C17SCV	Ginsenoside Rg7	C_36_H_60_O_9_	636.4237	Leaf	[39]
136	C17SCV	Ginsenoside Rh6	C_36_H_62_O_11_	670.4292	Fruit, leaf	[39]
137	C17SCV	Ginsenoside Ki	C_36_H_62_O_10_	654.4343	Leaf	[39]
138	C17SCV	Ginsenoside Km	C_36_H_62_O_10_	654.4343	Leaf	[84]
139	C17SCV	Ginsenoside Rh9	C_36_H_60_O_9_	636.4237	Leaf	[39]
140	C17SCV	12,23-Epoxyginsenoside Rg1	C_42_H_70_O_14_	798.4766	Leaf	[85]
141	C17SCV	Ginsenoside Rh7	C_36_H_60_O_9_	636.4237	Leaf	[39]
142	C17SCV	Ginsenoside Rh8	C_36_H_60_O_9_	636.4237	Leaf	[39]
143	C17SCV	Hexanordammaran	C_24_H_40_O_4_	392.2927	Leaf	[86]
144	C17SCV	Floralginsenoside A	C_42_H_72_O_16_	832.4820	Flower	[87]
145	C17SCV	Ginsenoside La	C_42_H_70_O_13_	782.4816	Leaf	[35]
146	C17SCV	Vinaginsenoside R4	C_48_H_82_O_19_	962.5450	Root, fruit, leaf	[16]
147	C17SCV	Ginsenoside Rk1	C_42_H_70_O_12_	766.4867	Root(steamed), fruit, leaf	[16]
148	C17SCV	Floralginsenoside H	C_50_H_84_O_21_	1020.5505	Flower	[88]
149	C17SCV	Floralginsenoside Tc	C_53_H_90_O_24_	1110.5822	Flower	[89]
150	C17SCV	Floralginsenoside Td	C_53_H_90_O_24_	1110.5822	Flower	[84]
151	C17SCV	Ginsenoside I	C_48_H_82_O_20_	978.5400	Flower	[90]
152	C17SCV	Ginsenoside II	C_48_H_82_O_20_	978.5400	Flower	[90]
153	C17SCV	Floralginsenoside C	C_41_H_70_O_15_	802.4715	Flower	[74]
154	C17SCV	Floralginsenoside J	C_48_H_82_O_20_	978.5400	Flower	[88]
155	C17SCV	Floralginsenoside Ka	C_36_H_62_O_11_	670.4292	Flower	[91]
156	C17SCV	Floralginsenoside La	C_48_H_82_O_19_	962.5450	Flower	[88]
157	C17SCV	Floralginsenoside Lb	C_48_H_82_O_19_	962.5450	Flower	[88]
158	C17SCV	Floralginsenoside Ta	C_36_H_60_O_10_	652.4187	Flower	[89]
159	C17SCV	Floralginsenoside E	C_42_H_72_O_15_	816.4871	Flower	[74]
160	C17SCV	Floralginsenoside F	C_42_H_72_O_15_	816.4871	Flower	[74]
161	C17SCV	Floralginsenoside G	C_50_H_84_O_21_	1020.5505	Flower	[88]
162	C17SCV	Floralginsenoside K	C_48_H_82_O_21_	994.5349	Flower	[88]
163	C17SCV	Floralginsenoside O	C_53_H_90_O_22_	1078.5924	Flower	[73]
164	C17SCV	Floralginsenoside B	C_42_H_72_O_16_	832.4820	Flower	[74]
165	C17SCV	Floralginsenoside D	C_41_H_70_O_15_	802.4715	Flower	[74]
166	C17SCV	Floralginsenoside I	C_48_H_82_O_20_	978.5400	Flower	[88]
167	C17SCV	Floralginsenoside Kb	C_45_H_76_O_19_	920.4981	Flower	[91]
168	C17SCV	Floralginsenoside Kc	C_45_H_76_O_20_	936.4930	Flower	[91]
169	C17SCV	Floralginsenoside Tb	C_35_H_62_O_11_	658.4292	Flower	[89]
170	C17SCV	Ginsenoside III	C_48_H_80_O_19_	960.5294	Flower	[92]

^1^ OA: Oleanolic acid; PPD: Protopanaxadiol; PPT: Protopanaxatriol; C17SCV: C17 side-chain varied.

**Table 2:** Isomers of 170 ginseng saponins.

No.	Formula	Molecular Mass	No. of Isomers	No.	Formula	Molecular Mass	No. of Isomers
1	C_24_H_40_O_4_	392.2927	1	36	C_46_H_76_O_15_	868.5184	2
2	C_30_H_50_O_2_	442.3811	2	37	C_47_H_80_O_17_	916.5396	6
3	C_30_H_50_O_3_	458.3760	2	38	C_47_H_80_O_18_	932.5345	2
4	C_30_H_52_O_3_	460.3916	2	39	C_48_H_78_O_18_	942.5188	1
5	C_30_H_52_O_4_	476.3866	2	40	C_48_H_80_O_19_	960.5294	1
6	C_30_H_54_O_4_	478.4022	1	41	C_48_H_82_O_18_	946.5501	3
7	C_30_H_54_O_5_	494.3971	1	42	C_48_H_82_O_19_	962.5450	9
8	C_35_H_62_O_11_	658.4292	1	43	C_48_H_82_O_20_	978.5400	4
9	C_36_H_60_O_10_	652.4187	1	44	C_48_H_82_O_21_	994.5349	1
10	C_36_H_60_O_7_	604.4339	3	45	C_49_H_78_O_19_	970.5137	1
11	C_36_H_60_O_8_	620.4288	2	46	C_49_H_80_O_18_	956.5345	1
12	C_36_H_60_O_9_	636.4237	5	47	C_50_H_84_O_19_	988.5607	3
13	C_36_H_62_O_10_	654.4343	3	48	C_50_H_84_O_21_	1020.5505	2
14	C_36_H_62_O_11_	670.4292	2	49	C_51_H_84_O_21_	1032.5505	8
15	C_36_H_62_O_8_	622.4445	4	50	C_53_H_90_O_22_	1078.5924	6
16	C_36_H_62_O_9_	638.4394	4	51	C_53_H_90_O_23_	1094.5873	1
17	C_37_H_62_O_10_	666.4343	1	52	C_53_H_90_O_24_	1110.5822	2
18	C_38_H_62_O_9_	662.4394	2	53	C_54_H_87_O_24_	1119.5587	1
19	C_41_H_70_O_12_	754.4867	2	54	C_54_H_92_O_22_	1092.6080	1
20	C_41_H_70_O_13_	770.4816	7	55	C_54_H_92_O_23_	1108.6029	1
21	C_41_H_70_O_15_	802.4715	2	56	C_54_H_92_O_24_	1124.5979	2
22	C_42_H_69_O_13_	781.4738	1	57	C_55_H_92_O_23_	1120.6029	4
23	C_42_H_70_O_12_	766.4867	6	58	C_56_H_92_O_25_	1164.5928	6
24	C_42_H_70_O_13_	782.4816	4	59	C_56_H_96_O_24_	1152.6292	1
25	C_42_H_70_O_14_	798.4766	2	60	C_57_H_93_O_23_	1145.6108	1
26	C_42_H_72_O_13_	784.4973	5	61	C_57_H_94_O_23_	1146.6186	2
27	C_42_H_72_O_14_	800.4922	5	62	C_57_H_94_O_25_	1178.6084	1
28	C_42_H_72_O_15_	816.4871	4	63	C_58_H_96_O_24_	1176.6292	2
29	C_42_H_72_O_16_	832.4820	2	64	C_58_H_98_O_26_	1210.6346	2
30	C_44_H_72_O_13_	808.4973	2	65	C_59_H_100_O_27_	1240.6452	1
31	C_44_H_74_O_14_	826.5079	2	66	C_60_H_99_O_27_	1251.6373	1
32	C_44_H_74_O_15_	842.5028	2	67	C_62_H_102_O_27_	1278.6608	1
33	C_45_H_74_O_17_	886.4926	1	68	C_62_H_102_O_30_	1326.6456	2
34	C_45_H_76_O_19_	920.4981	1	69	C_65_H_100_O_21_	1216.6757	1
35	C_45_H_76_O_20_	936.4930	1				

## References

[1] Wang H.P., Zhang Y.B., Yang X.W., Yang X.B., Xu W., Xu F., Cai S.Q., Wang Y.P., Xu Y.H., Zang L.X. (2016). High-performance liquid chromatography with diode array detector and electrospray ionization ion trap time-of-flight tandem mass spectrometry to evaluate ginseng roots and rhizomes from different regions. Molecules.

[2] Yang W.Z., Hu Y., Wu W.Y., Ye M., Guo D.A. (2014). Saponins in the *genus Panax* L. (Araliaceae): A systematic review of their chemical diversity. Phytochemistry.

[3] Qi L.W., Wang C.Z., Yuan C.S. (2011). ChemInform abstract: Isolation and analysis of *ginseng*: Advances and challenges. Nat. Prod. Rep..

[4] Shin B.H., Kwon S.W., Jeong Hill Park J.H. (2015). Chemical diversity of ginseng saponins from *Panax ginseng*. J. Ginseng Res..

[5] Shi Z.Y., Zeng J.Z., Wong A.S.T. (2019). Chemical structures and pharmacological profiles of ginseng saponins. Molecules.

[6] Lim K.H., Cho J.Y., Kim B., Bae B.S., Kim J.H. (2014). Red ginseng (*panax ginseng*) decreases isoproterenol-induced cardiac injury via antioxidant properties in porcine. J. Med. Food.

[7] Chung S.I., Kang M.Y., Lee S.C. (2016). In vitro and in vivo antioxidant activity of aged ginseng *(panax ginseng*). Prev. Nutr. Food Sci..

[8] Angelova N., Kong H.W., Heijden R., Yang S.Y., Choi Y., Kim H., Wang M., Hankemeier T., van der Greef J., Xu G. (2008). Recent methodology in the phytochemical analysis of ginseng. Phytochem. Anal..

[9] Woo H.C., Shin B.K., Cho I., Koo H., Kim M., Han J. (2011). Anti-obesity Effect of Carbon Dioxide Supercritical Fluid Extracts of *Panax Ginseng* C. A. Meyer. J. Korean Soc. Appl. BI..

[10] Kim Y.J., Zhang D.Z., Yang D.C. (2015). Biosynthesis and biotechnological production of ginsenosides. Biotechnol. Adv..

[11] Lü J.M., Yao Q., Chen C. (2009). Ginseng compounds: An update on their molecular mechanisms and medical applications. Curr. Vasc. Pharmacol..

[12] Chen X.P., Lin Y., Hu Y., Liu C.X., Lan K., Jia W. (2015). Phytochemistry, Metabolism, and Metabolomics of Ginseng. Chin. Herb. Med..

[13] Yang X.W. (2016). Triterpenoids in *Panax ginseng*. Mod. Chin. Med..

[14] Wang H.P., Zhang Y.B., Yang X.W., Zhao D.Q., Wang Y.P. (2015). Rapid characterization of ginsenosides in the roots and rhizomes of *panax ginseng* by UPLC-DAD-QTOF-MS/MS and simultaneous determination of 19 ginsenosides by HPLC-ESI-MS. J. Ginseng Res..

[15] Mao Q., Bai M., Xu J.D., Kong M., Zhu L.Y., Zhu H., Wang Q., Li S.L. (2014). Discrimination of leaves of *Panax ginseng* and *P. quinquefolius* by ultra high performance liquid chromatography quadrupole/time-of-flight mass spectrometry based metabolomics approach. J. Pharm. Biomed. Anal..

[16] Lee J.W., Choi B.R., Kim Y.C., Choi D., Lee Y.S., Kim G.S., Baek N.I., Kim S.Y., Lee D.Y. (2017). Comprehensive profiling and quantification of ginsenosides in the root, stem, leaf, and berry of *Panax ginseng* by UPLC-QTOF/MS. Molecules.

[17] Chu S.F., Zhang J.T. (2009). New achievements in ginseng research and its future prospects. Chin. J. Integr. Med..

[18] Jia L., Zhao Y., Liang X.J. (2009). Current evaluation of the millennium phytomedicine-ginseng (II): Collected chemical entities, modern pharmacology, and clinical applications emanated from traditional chinese medicine. Curr. Med. Chem..

[19] Garriques S.S. (1854). On panaquilon, a new vegetable substance. Am. J. Pharm..

[20] Shibata S., Tanaka O., Sado M., Tsushima S. (1963). On genuine sapogenin of ginseng. Tetrahedron Lett..

[21] Sanada S., Kondo N., Shoji J., Tanaka O., Shibata S. (1974). Studies on the saponins of ginseng. I. structures of Ginsenoside-Ro, -Rb1, -Rb2, -Rc and -Rd. Chem. Pharm. Bull..

[22] Sanada S., Kondo N., Shoji J., Tanaka O., Shibata S. (1974). Studies on the saponins of ginseng. II. Structures of ginsenoside Re, -Rf and -Rg2. Chem. Pharm. Bull..

[23] Nagai Y., Tanaka O., Shibata S. (1971). Chemical studies on the oriental plant drugs—XXIV: Structure of ginsenoside-Rg1, a neutral saponin of ginseng root. Tetrahedron.

[24] Shibata Y., Nozaki T., Higashi T., Sanada S., Shoji J. (1976). Saponins of leaves of *Panax ginseng* C.A. Meyer. Chem. Pharm. Bull..

[25] Yahara S. (1976). Sapinins of bads and flowers of *Panax ginseng* C. A. Meyer isolation of ginsenside Rd, Re, Rg1. Chem. Pharm. Bull..

[26] Sanada S., Shoji J. (1978). Studies on the Saponins of ginseng III, Structures of ginsenoside-Rb3 and 20-Glucoginsenoside-Rf. Chem. Pharm. Bull..

[27] Cai P. (1982). Isolation and identification of ginsenosides in ginseng leaves of Jilin Province. Chinese Pharm. Bull..

[28] Shao C.J., Xu J.D., Jiang X.K., Cheng G.R. (1984). Studies on the chemical constituents of flower-buds of *Panax*
*ginseng* C.A. Meyer in Jilin(I). J. Jilin Univ. Med. Ed..

[29] Shao C.J., Xu J.D. (1984). Chemical studies on the tetracyclic tirterpenic saponins in flower-buds of *Panax*
*ginseng* C.A. Meyer. Chem. J. Chinese Unvi..

[30] Xu S.X., Wang N.L., Shen M., Lu X.K. (1986). Chemical constituents of saponins of stems and leaves of *Panax*
*ginseng* C.A. Meyer. Acta Bot. Sin..

[31] Chen Y.J., Xu S.X., Ma Q.F. (1987). Study on the new minor new constituents of ginseng leaves. Acta Pharm. Sin..

[32] Zhang S., Chen Y., Cui C., He G., Xu S., Pei Y., Yao X., Zhu T. (1989). A new minor saponin from the leaves of *Panax ginseng* C. A. Meyer. Acta Pharm. Sin..

[33] Chen Y., Zhang S., Wang Z., Lu Y., Xu S., Yao X., Cui C., Tezuka Y., Kikuchi T., Ogihara Y. (1990). Isolation and elucidation of a new minor saponin from the leaves of *Panax ginseng* C.A. Meyer. Acta Pharm. Sin..

[34] Kim D.S., Chang Y.J., Zedk U., Zhao P., Lin Y.Q., Yang C.R. (1995). Dammarane saponins from *Panax ginseng*. Phytochemistry.

[35] Dou D., Wen Y., Weng M., Pei Y., Chen Y. (1997). Studies on the Minor Saponins from Leaves of *Panax ginseng* C.A.Meyer. China J. Chin. Mat. Med..

[36] Dou D.Q., Hou W.B., Chen Y.J. (1998). Studies of the Characteristic Constituents of Chinese Ginseng and American Ginseng. Planta Med..

[37] Dou D.Q., Chen Y.J., Liang L.H., Pang F.G., Shimizu N., Takeda T. (2001). Six New Dammarane-type Triterpene Saponins from the Leaves of *Panax ginseng*. Chem. Pharm. Bull..

[38] Dou D.Q., Ren J., Chen Y., Pei Y.P., Chen Y.J. (2003). Study on the chemical constituents of the roots of commercial ginseng. China J. Chin. Mater. Med..

[39] Liu C.X., Xiao P.G. (1992). Recent advances on ginseng research in China. J. Ethnopharmacol..

[40] Nagasawa T., Oura H., Choi J.H., Bae J.W. Application of high-per-formance liquid chromatography to the isolation of ginsenosides from ginseng saponins. Proceedings of the Ginseng society Conference.

[41] Shibata S., Tanaka O., Ando T., Sado M., Tsushima S., Ohsawa T. (1966). chemical studies on oriental plant drugs. XIV. Protopanaxadiol, a genuine sapogenin of ginseng saponins. Chem. Pharm. Bull..

[42] Liu F.F., Zhang A.H., Lei F.J., Xiu Y.H., Zhang L.X. (2018). Inhibiting effects of total ginsenosides of ginseng stems and leaves against *Fusarium solani* and their antibacterial mechanism. J. Jilin Agric. Univ..

[43] Zhang A.H., Tan S.Q., Zhao Y., Feng J.L., Zhang L.X. (2015). Effects of total ginsenosides on the feeding behavior and two enzymes activities of *Mythimna separata* (Walker) Larvae. Evid. Based Complementary Altern. Med..

[44] Zhang H., Lu Z., Tan G.T., Qiu S., Farnsworth N.R., Pezzuto J.M., Fong H.H.S. (2002). Polyacetylene ginsenoside-Ro, a novel triterpene saponin from *Panax ginseng*. Tetrahedron Lett..

[45] Zhou Q.L., Xu W., Yang X.W. (2016). Chemical constituents of Chinese red ginseng. China J. Chin. Mater. Med..

[46] Lee D.G., Lee J., Yang S., Kim K.T., Lee S. (2015). Identification of dammarane-type triterpenoid saponins from the root of *Panax ginseng*. Nat. Prod. Sci..

[47] Yang W.Z., Ye M., Qiao X., Liu C.F., Miao W.J., Bo T., Tao H.Y., Guo D.A. (2012). A strategy for efficient discovery of new natural compounds by integrating orthogonal column chromatography and liquid chromatography/mass spectrometry analysis: Its application in *Panax ginseng*, *Panax quinquefolium* and *Panax notoginseng* to characterize 437 potential new ginsenosides. Anal. Chim. Acta.

[48] Besso H., Kasai R., Saruwatari Y., Fuwa T., Tanaka O. (1982). Ginsenoside-Ra1 and ginsenoside-Ra2, new dammarane-saponins of ginseng roots. Chem. Pharm. Bull..

[49] Matsuura H., Kasai R., Tanaka O., Saruwatari Y., Kunihiro K., Fuwa T. (1984). Further studies on dammarane-saponins of ginseng roots. Chem. Pharm. Bull..

[50] Kasai R., Besso H., Tanaka O., Saruwatari Y., Fuwa T. (1983). Saponins of red ginseng. Chem. Pharm. Bull..

[51] Ruan C.C., Liu Z., Li X., Liu X., Wang L.J., Pan H.Y., Zheng Y.N., Sun G.Z., Zhang Y., Zhang L.X. (2010). Isolation and Characterization of a New Ginsenoside from the Fresh Root of *Panax Ginseng*. Molecules.

[52] Sun G.Z., Li X.G., Liu Z., Wang J.Y., Zheng Y.N., Yang X.W. (2007). Isolation and structure characterization of malonylnotoginsenoside-R4 from the root of *Panax ginseng*. Chem. J. Chin. Univ..

[53] Zhu G.Y., Li Y.W., Hau D., Jiang Z.H., Yu Z.L., Fong W.F. (2011). Acylated Protopanaxadiol-Type Ginsenosides from the Root of *Panax ginseng*. Chem. Biodivers..

[54] Kaku T., Kawashima Y. (1980). Isolation and characterization of ginsenoside Rg2, 20R-prosapogenin, 20S-prosapogenin and D20 -prosapogenin. Chemical studies on saponins of *Panax ginseng* C.A. Meyer. Third report. Arzneimittelforschung.

[55] Baek N.I., Kim J.M., Park J.H., Ryu J.H., Kim D.S., Lee Y.H., Park J.D., Kim S.I. (1997). Ginsenoside Rs3, a genuine dammarane-glycoside from Korean red ginseng. Arch. Pharm. Res..

[56] Chen W., Balan P., Popovich D. (2020). Ginsenosides analysis of New Zealand grown forest *Panax ginseng* using LC-QTOF-MS/MS. J. Ginseng Res..

[57] Tao L., Li K., Li D., Gong X. (2018). Saponin Constituents from Fruits of *Panax ginseng*. Mod. Chin. Med..

[58] Wang W., Rayburn E., Hill D., Wang H., Zhang R. (2007). In vitro anti-cancer activity and structure-activity relationships of natural products isolated from fruits of *Panax ginseng*. Cancer Chemother. Pharmacol..

[59] Xu M., Zhan Z.J., Zhang X.Y. (2007). Study on the chemical constituents of ginseng fruit. Zhong Cao Yao.

[60] Lee M., Seo H., Singh D., Lee S.J., Lee C. (2019). Unraveling dynamic metabolomes underlying different maturation stages of berries harvested from *Panax ginseng*. J. Ginseng Res..

[61] Dou D.Q., Chen Y.J., Meng Z.Y., Wen Y., Pei Y.P., Xu S.X., Yao X.S., Kawai H., Fukushima H., Murkami Y. (1996). Two Minor Saponins from Leaves of *Panax ginseng* C. A. Meyer. J. Chin. Pharm. Sci..

[62] Siddiqi M., Siddiqi M.Z., Sungeun A., Kang S., Kim Y.J., Natarajan S., Yang D.U., Yang D.C. (2013). Ginseng saponins and the treatment of osteoporosis: Mini literature review. J. Ginseng Res..

[63] Kang K., Ham J., Kim Y.J., Park J., Cho E.J., Yamabe N. (2013). Heat-processed *Panax ginseng* and diabetic renal damage: Active components and action mechanism. J. Ginseng Res..

[64] Lee S., Kim M.G., Ko S., Kim H.K., Leem K., Kim Y.J. (2014). Protective effect of ginsenoside Re on acute gastric mucosal lesion induced by compound **48**/**80**. J. Ginseng Res..

[65] Lee C., Kim J.H. (2014). A review on the medicinal potentials of ginseng and ginsenosides on cardiovascular diseases. J. Ginseng Res..

[66] Sun M., Che Y., Liu Z. (2015). A tractable method for the preparation of the ginsenoside compounds O and Mc1. Anal. Methods.

[67] Li F., Li Q., Wang J., Lv C., Song D., Liu P., Zhang D., Lu J. (2016). Chemical and bioactive comparison of flowers of *Panax ginseng* Meyer, *Panax quinquefolius* L. and *Panax notoginseng* Burk. J. Ginseng Res..

[68] Qiu S., Yang W.Z., Yao C., Shi X.J., Li J.Y., Lou Y., Duan Y.N., Wu W.Y., Guo D.A. (2017). Malonylginsenosides with potential antidiabetic activities from the flower buds of *Panax ginseng*. J. Nat. Prod..

[69] Kitagawa I., Yoshikawa M., Yoshihara M., Hayashi T., Taniyama T. (1983). Chemical Studies on Crude Drug Precession. I. On the Constituents of Ginseng Radix Rubra (1). Yakugaku Zasshi..

[70] Zhu G.Y., Li Y.W., Hau D., Jiang Z.H., Yu Z.L., Fong W.F. (2011). Protopanaxatriol-Type ginsenosides from the root of *Panax ginseng*. J. Agric. Food Chem..

[71] Dou D.Q., Wen Y.E., Pei Y., Yao X.S., Chen Y., Kawai H., Fukushima H. (1996). Ginsenoside-Ia: A Novel Minor Saponin from the Leaves of *Panax ginseng*. Planta Med..

[72] Ma H.Y., Gao H., Huang J., Sun B.H., Yang B. (2012). Three new triterpenoids from *Panax ginseng* exhibit cytotoxicity against human A549 and Hep-3B cell lines. J. Nat. Med..

[73] Yoshikawa M., Sugimoto S., Nakamura S., Sakumae H., Matsuda H. (2007). Medicinal Flowers. XVI. New Dammarane-Type Triterpene Tetraglycosides and Gastroprotective Principles from Flower Buds of *Panax ginseng*. Chem. Pharm. Bull..

[74] Yoshikawa M., Sugimoto S., Nakamura S., Matsuda H. (2007). Medicinal Flowers. XI. Structures of new dammarane-type triterpene diglycosides with hydroperoxide group from flower buds of *Panax ginseng*. Chem. Pharm. Bull..

[75] Park I., Han S., Kim J., Piao L., Kwon S., Kim N., Kang T., Park M., Park J. (2002). Four new acetylated ginsenosides from processed ginseng (sun ginseng). Arch. Pharm. Res..

[76] Park I., Kim N., Han S., Kim J., Kwon S., Kim H.J., Park M., Park J. (2002). Three new dammarane glycosides from heat processed ginseng. Arch. Pharm. Res..

[77] Lee S., Shon H., Choi C.-S., Tran Manh H., Min B., Bae K. (2009). Ginsenosides from Heat Processed Ginseng. Chem. Pharm. Bull..

[78] Ryu J., Park J.-H., Eun J.-H., Jung J.-H., Sohn D. (1997). A dammarane glycoside from Korean red ginseng. Phytochemistry.

[79] Lee S., Oh J., Na M. (2013). Updating chemical profiling of red ginseng via the elucidation of two geometric isomers of ginsenosides Rg9 and Rg10. Food Chem..

[80] Cho J.G., Lee D.Y., Shrestha S., Lee S.K., Kang H.M., Son S.H., Yang D.C., Baek N.I. (2013). Three New Ginsenosides from the Heat-Processed Roots of *Panax ginseng*. Chem. Nat. Compd..

[81] Wang J.Y., Li X.G., Zheng Y.N., Yang X. (2005). Isoginsenoside-Rh3, a new triterpenoid saponin from the fruits of *Panax ginseng* C. A. Mey. J. Asian Nat. Prod. Res..

[82] Yu M., Zhao Y.Q. (2004). Chemical Study on triterpenoids in ginseng fruit. Zhong Cao Yao.

[83] Han W., Lu S., Wen H., Xu L., Jin J., Tang S. (2018). Chemical constituents from fruit pedicels of *Panax ginseng*. Zhong Cao Yao.

[84] Nguyen H., Song G., Kim J.A., Hyun J.H., Kang H.K., Kim Y.H. (2009). Dammarane-type saponins from the flower buds of *Panax ginseng* and their effects on human leukemia cells. Bioorganic Med. Chem. Lett..

[85] Wang L., Wu Z., Gao H., Huang J., Sun B., Wu L. (2008). A new compound with cytotoxic activities from the leaves of *Panax ginseng* C.A. Meyer. Chin. Chem. Lett.

[86] Wu L.J., Wang L.B., Gao H., Wu B., Song X.M., Tang Z.S. (2008). A new compound from the leaves of *Panax ginseng*. Fitoterapia.

[87] Lee D.Y., Lee J., Jeong Y.T., Byun G.H., Kim J.H. (2017). Melanogenesis inhibition activity of floralginsenoside A from *panax ginseng* berry. J. Ginseng Res..

[88] Nakamura S., Sugimoto S., Matsuda H., Yoshikawa M. (2007). Structures of dammarane type triterpene triglycosides from the flower buds of *Panax ginseng*. Heterocycles.

[89] Tung N.H., Song G.Y., Woo S.H., Hyun J.W., Koh Y.S., Kang H.K. (2012). Ginsenosides from the leaves and flower buds of *panax ginseng* and their pharmacological effects. Curr. Bioact. Compd..

[90] Qiu F., Ma Z., Xu S.X., Yao X.S., Che C.T., Chen Y.J. (2001). A Pair of 24-hydroperoxyl Epimeric Dammarane Saponins from Flower-buds of *Panax Ginseng*. J. Asian Nat. Prod. Res..

[91] Nguyen H.T., Song G.Y., Nhiem N.X., Ding Y., Tai B.H., Jin L.G., Lim C.M., Hyun J.W., Park C.J., Kang H.K. (2010). Dammarane-type saponins from the flower buds of *Panax ginseng* and their intracellular radical scavenging capacity. J. Agric. Food chem..

[92] Qiu F., Ma Z., Xu S., Yao X.S., Chen Y., Che Z. (1998). Studies on Dammarane-Type Saponins in the Flower-Buds of *Panax ginseng* C.A. Meyer. J. Asian Nat. Prod. Res..

[93] Fuzzati N. (2005). Analysis methods of ginsenosides. J. Chromatogr. B.

[94] Baek S.H., Bae O.N., Park J. (2012). Recent Methodology in Ginseng Analysis. J. Ginseng Res..

[95] Cui J., Björkhem I., Eneroth P. (1997). Gas chromatographic-mass spectrometric determination of 20(*S*)-protopanaxadiol and 20(*S*)-protopanaxatriol for study on human urinary excretion of ginsenosides after ingestion of ginseng preparations. J. Chromatogr. B.

[96] Kim B.Y., Lee M., Cho K., Park J., Park M. (1992). Analysis of ginseng saponins by HPLC with photoreduction fluorescence detection. Arch. Pharmacol. Res..

[97] Van Breemen R., Huang C.-R., Lu Z.-Z., Rimando A., Fong H., Fitzloff J. (1995). Electrospray Liquid Chromatography/Mass Spectrometry of Ginsenosides. Anal. Chem..

[98] Wang X., Sakuma T., Asafu-Adjaye E., Shiu G. (1999). Determination of ginsenosides in plant extracts from *panax ginseng* and *Panax quinquefolius* L. by LC/MS/MS. Anal. Chem..

[99] Chan D., But P.P.H., Cheng S.W., Kwok I.M.Y., Lau F.W., Xu H.X. (2000). Differentiation and authentication of *Panax ginseng*, *Panax quinquefolius*, and ginseng products by using HPLC/MS. Anal. Chem..

[100] Sloley B., Lin Y.C., Ridgway D., Semple H., Tam Y., Coutts R., Löbenberg R., Tam-Zaman N. (2006). A method for the analysis of ginsenosides, malonyl ginsenosides, and hydrolyzed ginsenosides using high-performance liquid chromatography with ultraviolet and positive mode electrospray ionization mass spectrometric detection. J. AOAC Int..

[101] Park H.W., In G., Kim J.H., Cho B.G., Han G.H., Chang I.M. (2014). Metabolomic approach for discrimination of processed ginseng genus (*Panax ginseng* and *Panax quinquefolius*) using UPLC-QTOF MS. J. Ginseng Res..

[102] Popovich D., Kitts D. (2004). Generation of ginsenosides Rg3 and Rh2 from North American ginseng. Phytochemistry.

[103] Sun B.S., Xu M.Y., Li Z., Wang Y.B., Sung C.K. (2012). UPLC-Q-TOF-MS/MS Analysis for steaming times-dependent profiling of steamed *Panax quinquefolius* and its ginsenosides transformations induced by repetitious steaming. J. Ginseng Res..

[104] Pascoe R., Foley J., Gusev A. (2002). Reduction in Matrix-Related Signal Suppression Effects in Electrospray Ionization Mass Spectrometry Using On-Line Two-Dimensional Liquid Chromatography. Anal. Chem..

[105] Ma X., Xiao H., Liang X. (2006). Identification of Ginsenosides in Panax quinquefolium by LC-MS. Chromatographia.

[106] Xu W., Qiu X.-H., Zhang J., Zhu D.Y., Yang Y.M., Lu C.J. (2012). Analysis of saponins in *Panax notoginseng* by UPLC-LTQ-Orbitrap MS/MS. Acta Pharm. Sin..

[107] Li W., Gu C., Zhang H., Awang D.V.C., Breemen R.B.V. (2000). Use of High-Performance Liquid Chromatography−Tandem Mass Spectrometry To Distinguish *Panax ginseng* C.A. Meyer (Asian Ginseng) and *Panax quinquefolius* L. (North American Ginseng). Anal. Chem..

[108] Xü Z.X., Xiao H.B., Wang J.N., Liang X.M. (2000). Analysis of ginsenosides by high performance liquid chromatography/mass spectrometry/mass spectrometry (lc/ms/ms). Se Pu.

[109] Chen Y., Zhao Z., Chen H., Qin M., Liang Z. (2014). Chemical Differentiation and Quality Evaluation of Commercial Asian and American Ginsengs based on a UHPLC–QTOF/MS/MS Metabolomics Approach. Phytochem. Anal..

[110] Zhang H.M., Li S.L., Zhang H., Wang Y., Zhao Z.L., Chen S.L., Xu H.X. (2012). Holistic quality evaluation of commercial white and red ginseng using a UPLC-QTOF-MS/MS-based metabolomics approach. J. Pharm. Biomed. Anal..

[111] Zhang X.X., Wang H.P., Yang Y., Du M.B., Mao S., Chen C., Liu Y.X., Li S.J. (2015). Rapid analysis of ginsenosides in dried fresh Ginseng by ultra -performance liquid chromatography with quadrupole time-of-flight mass spectrometry. China Medical Herald..

[112] Shi X.J., Yang W.Z., Qiu S., Yao C., Shen Y., Pan H.Q., Bi Q.R., Yang M., Wu W.Y., Guo D.A. (2016). An in-source multiple collision-neutral loss filtering based nontargeted metabolomics approach for the comprehensive analysis of malonyl-ginsenosides from *Panax ginseng*, *P. quinquefolius,* and *P. notoginseng*. Anal. Chim. Acta.

[113] Shi X., Yang W., Huang Y., Hou J., Qiu S., Yao C., Feng Z., Wei W., Wu W., Guo D. (2018). Direct screening of malonylginsenosides from nine Ginseng extracts by an untargeted profiling strategy incorporating in-source collision-induced dissociation, mass tag, and neutral loss scan on a hybrid linear ion-trap/Orbitrap mass spectrometer coupled to ultra-high performance liquid chromatography. J. Chromatogr. A.

[114] Zhong W., Dai Y.L., Li X.Y., Wang Y.B., Liu S.Y. (2015). Analysis of chemical differences in red ginseng by Uplc-Q-Orbitrap mass spectrometry. J. Chin. Mass Spectrom. Soc..

